# Assessment of Cardiac Remodeling—A Chance for Novel Cardiac Biomarkers?

**DOI:** 10.3390/jcm9072087

**Published:** 2020-07-03

**Authors:** Peter Jirak, Moritz Mirna, Bernhard Wernly, Vera Paar, Uta C. Hoppe, Michael Lichtenauer

**Affiliations:** Department of Internal Medicine II, Division of Cardiology, Paracelsus Medical University Salzburg, 5020 Salzburg, Austria; p.jirak@salk.at (P.J.); m.mirna@salk.at (M.M.); b.wernly@salk.at (B.W.); v.paar@salk.at (V.P.); u.hoppe@salk.at (U.C.H.)

## 1. Background

Biomarkers are defined as “cellular, biochemical or molecular alterations that are measurable in biological media such as human tissues, cells, or fluids”, providing “biological characteristics that can be objectively measured and evaluated as an indicator of normal biological processes, pathogenic processes, or pharmacological responses to a therapeutic intervention “according to Hulka et al. as well as Naylor et al. [1,2]. Depending on their respective role in physiologic and pathophysiologic processes, biomarkers can be used for different purposes such as disease diagnosis, risk stratification, screening, as well as prognosis [2]. In recent decades, biomarkers have gained major clinical significance, especially in the cardiovascular field. Above all, the introduction of natriuretic peptides and highly sensitive troponin assays have led to significant facilitation and improvement in clinical practice. 

However, while these markers represent indispensable diagnostic tools in clinical routines, their prognostic impact remains limited. Accordingly, the evaluation of prognosis remains a clinical challenge, even to date. In contrast to the aforementioned markers, novel biomarkers targeting the critical factors for prognosis and outcomes in cardiovascular disease, cardiac fibrosis, and remodeling could be of additional value on this account [3,4]. Cardiac remodeling: The term cardiac remodeling describes changes in the size; mass; geometry; and, consequently, function of the heart in response to acute and chronic myocardial damage [5]. While acute myocardial damage is usually induced by ischemic processes such as myocardial infarction, chronic damage comprises inflammatory processes, dysregulated metabolic pathways, toxic damage, as well as a chronic increase in cardiac strain [6,7,8]. 

Interestingly, in contrast to natriuretic peptides as well as troponin, most novel heart failure biomarkers do not provide a comparable amount of organ specificity [9,10]. However, due to their involvement in multiple pathophysiologic processes, novel cardiac biomarkers represent promising tools to refine the assessment of cardiac remodeling and fibrosis and thus also of prognosis [4,11].

## 2. sST2

The most promising marker on this regard represents soluble suppression of tumorigenicity 2 (sST2), which has also found entrance into current guidelines to some extent. sST2 represents a versatile marker, predominantly used in heart failure patients [12]. sST2 was shown to be elevated in acute and chronic heart failure as well as in acute coronary syndrome [13,14]. Besides, elevated levels have also been reported in pulmonary hypertension and peripheral artery disease, emphasizing its involvement in different disease entities [15,16]. 

Regarding its molecular background, two different isoforms have been identified, a soluble form (sST2) and a membrane-bound form (ST2L) [17]. Interleukin-33 (IL-33) represents the only known ligand for ST2 and is responsible for the induction of cardioprotective effects by binding to the ST2L receptor [17]. Besides, IL-33 is also involved in immunomodulation through the secretion and the interaction of T helper 2 (TH2) cells, mast cells, group 2 innate lymphoid cells, (ILC2s) regulatory T (Treg) cells, TH1 cells, CD8+ T cells, and natural killer (NK) cells, among others, further elucidating the involvement of sST2 and ST2L in inflammatory processes [18]. On the other hand, sST2 can counteract the cardioprotective effects by acting as a decoy receptor for IL-33 [17]. Hence, an increase in sST2 results in a decrease of cardioprotective IL-33, consequently leading to cardiac damage and increased cardiac strain [17]. 

In short, sST2 incorporates different pathophysiological processes involved in cardiac remodeling and fibrosis such as inflammation and increased cardiac strain. Accordingly, sST2 represents a promising new marker in the assessment of prognosis of heart failure patients. sST2 was shown to predict all-cause mortality as well as cardiovascular mortality in chronic heart failure patients [19]. Additionally, sST2 was reported to predict mortality in acute heart failure [11]. With regards to therapy monitoring, an sST2 cut-off below 35-ng/mL was proposed to significantly improve outcomes in heart failure patients (Pres-ageassay, Critical Diagnostics, SanDiego, California, USA) [19]. 

## 3. microRNAs 

While sST2 has already found entrance into current guidelines, the field of microRNAs (miRNAs) is currently limited to investigative research, although previous trials have reported promising results regarding their diagnostic and therapeutic applicability in cardiovascular disease entities. MiRNAs comprise a group of small (19–24 nucleotides) ribonucleic acids (RNAs), which play a pivotal role in posttranslational gene silencing (PTGS) and hence regulation of protein synthesis [20,21]. In recent studies, several miRNAs were found to be involved in cardiac remodeling by promoting myocardial inflammation, as well as pro-fibrotic and -apoptotic pathways. Consequently, patients with acute and chronic heart failure show dysregulated plasma concentrations of various pathological miRNAs, which gives rise to novel diagnostic approaches in these patients. For example, Ovchinnikova et al. recently identified several significantly dysregulated miRNAs (miR-18a-5p, miR-26b-5p, miR-27a-3p, miR-30e-5p, miR-106a-5p, miR-199a-3p, and miR-652-3p) in acute heart failure (AHF), which were also associated with adverse outcomes in these patients [22]. Furthermore, another trial reported that a combination of miR-30c, miR-221, miR-328, and miR-375 could adequately discriminate heart failure with preserved ejection fraction (HFpEF) from heart failure with reduced ejection fraction (HFrEF) [23], which is to this extent not possible using conventional cardiovascular biomarkers [24]. Consequently, analysis of the miRNA expression pattern (“miRNome”) could provide substantial additional information in the clinical management of patients with HF in the future. 

Besides their application in diagnostics, miRNAs also constitute interesting targets for novel therapeutic approaches. Since myocardial inflammation and cardiac fibrosis are considered potentially reversible processes in the course of cardiac remodeling, miRNAs interacting with these pathways represent promising targets in the management of patients with HF. For example, miR-21 was found to enhance myocardial fibrosis by targeting the extracellular signal-regulated kinase (ERK)–mitogen-activated protein (MAP) kinase pathway, and silencing of miR-21 by synthetic antagonist significantly attenuated myocardial fibrosis in an animal model [25]. Since miR-21 was also found to be involved in myocardial inflammation by targeting T-cell development, it constitutes an interesting drug target in the management of HF and certainly warrants further investigation in future studies.

## 4. Conclusions

Despite the long time period since the establishment of natriuretic peptides and troponin in clinical practice, the evaluation of prognosis in cardiovascular disease remains challenging. The assessment of cardiac remodeling and fibrosis with the help of novel biomarkers represents a promising approach for a more sophisticated evaluation of prognosis and consequently also therapy guiding. In this regard, their versatility regarding their involvement in numerous different organ systems might be a considerable benefit over natriuretic peptides and troponin with regards to their prognostic value. As cardiac remodeling is strongly correlated with a worse prognosis in cardiovascular disease, the implementation of biomarkers addressing this issue holds great potential to improve outcomes further. 
